# High Throughput Measurement of γH2AX DSB Repair Kinetics in a Healthy Human Population

**DOI:** 10.1371/journal.pone.0121083

**Published:** 2015-03-20

**Authors:** Preety M. Sharma, Brian Ponnaiya, Maria Taveras, Igor Shuryak, Helen Turner, David J. Brenner

**Affiliations:** Center for Radiological Research, Columbia University, New York, New York, United States of America; Dana-Farber/Harvard Cancer Institute, UNITED STATES

## Abstract

The Columbia University RABiT (Rapid Automated Biodosimetry Tool) quantifies DNA damage using fingerstick volumes of blood. One RABiT protocol quantifies the total γ-H2AX fluorescence per nucleus, a measure of DNA double strand breaks (DSB) by an immunofluorescent assay at a single time point. Using the recently extended RABiT system, that assays the γ-H2AX repair kinetics at multiple time points, the present small scale study followed its kinetics post irradiation at 0.5 h, 2 h, 4 h, 7 h and 24 h in lymphocytes from 94 healthy adults. The lymphocytes were irradiated ex vivo with 4 Gy γ rays using an external Cs-137 source. The effect of age, gender, race, ethnicity, alcohol use on the endogenous and post irradiation total γ-H2AX protein yields at various time points were statistically analyzed. The endogenous γ-H2AX levels were influenced by age, race and alcohol use within Hispanics. In response to radiation, induction of γ-H2AX yields at 0.5 h and peak formation at 2 h were independent of age, gender, ethnicity except for race and alcohol use that delayed the peak to 4 h time point. Despite the shift in the peak observed, the γ-H2AX yields reached close to baseline at 24 h for all groups. Age and race affected the rate of progression of the DSB repair soon after the yields reached maximum. Finally we show a positive correlation between endogenous γ-H2AX levels with radiation induced γ-H2AX yields (RIY) (r=0.257, P=0.02) and a negative correlation with residuals (r=-0.521, P=<0.0001). A positive correlation was also observed between RIY and DNA repair rate (r=0.634, P<0.0001). Our findings suggest age, race, ethnicity and alcohol use influence DSB γ-H2AX repair kinetics as measured by RABiT immunofluorescent assay.

## Introduction

Following exposure to ionizing radiation, DNA double-strand breaks (DSBs) are produced at sites of DNA damage. One of the initial responses to the DSB is phosphorylation of histone H2AX protein that forms gamma-H2AX (γ-H2AX) foci within minutes at the DNA damage sites [1]. Each DSB site is believed to correspond to one microscopic γ-H2AX focus that rapidly forms and depending on functionality of the DNA repair response, the foci repair at different rates that is indicated by disappearance of foci at those sites. These foci repair over a period of two days [2–4] and can be followed microscopically by the immunofluorescent γ-H2AX assay that immunostains the phosphorylated H2AX histone represented as γ-H2AX. Both the intensity of the fluorescence at individual DSB sites and the number of γ-H2AX foci is directly proportional to the amount of DSB produced [5]. An ultra high throughput automated system, the RABiT (Rapid Automated BIodosimetry tool [6, 7] developed at the Center for High-Throughput Minimally Invasive Radiation Biodosimetry (CHTMIRB) estimates individual dose exposure using finger stick blood sample in an in-situ multi-well plate platform. This robotically based system is fully automated, and its high throughput is based on robotic handling, a multiwell plate platform, and rapid image analysis. One of the assays optimized in the system is the γ-H2AX assay [8] quantifying the DNA double strand breaks (DSB) [3, 4, 9] in isolated unstimulated lymphocytes. The non-proliferating lymphocytes are G0 phase cells that have low background γ-H2AX staining [10] which is advantageous for purpose of biodosimetry. These cells can be obtained in large numbers, in a short span of time from a small volume of blood and manipulated in few steps with minimal cell quality compromise. With the advantages of obtaining these cells with minimal invasive methods [8], high sensitivity and specificity of the γ-H2AX assay and ease of monitoring with automated microscopic techniques, this assay is well suited for biodosimetry in population studies [3, 4, 8, 11]. Inter-individual variability in γ-H2AX response [12, 13] is documented, although the effect of modulators such as genetic, environmental, age, gender, and other factors on radiation induced γ-H2AX formation, efficiency of DSB repair, and cellular radiosensitivity is not completely understood. Among factors modulating DNA repair, environmental factors such as smoking [14, 15], diet [16, 17] and factors such as age, gender [18–21] have been studied. At the cellular level, the kinetics of formation or loss of γ-H2AX foci may reflect the rate or efficiency of DSB repair [22–24]. The biphasic nature of DSB repair kinetics has been associated with different repair pathways that allow repair for a fast (initial few hours) and slow component (hours to days) of repair [25, 26]. Additionally, there is evidence that the DSBs assayed several hours after the initial radiation challenge that still remain unrepaired known as residual DNA damage, may be predictive of individual susceptibility to complex DNA lesions that can be lethal [2, 27, 28]. The rate of loss of foci and presence of residual foci has also been correlated with cellular radiosensitivity [29]. Further, it has been suggested that lower levels of initial DNA damage may be associated with a lower risk of severe late subcutaneous radiation effects [30].

It is thus implicit that study of factors affecting variability in DSB repair kinetics and residual levels are important for understanding individual radiation response and sensitivity and has been the subject of recent investigations. Recently a systematic evaluation of published population studies employing the γ-H2AX assay as a DSB repair and genomic instability marker was reported [31]. This study highlighted the technical and epidemiological heterogeneity and gaps in the knowledge of the impact of modulators such as environmental factors, genotype among others. Information about DNA repair response in humans of different age, gender, racial and ethnic origin is sparse.

The goals of the current small scale study are as follows: 1) to collect finger stick blood samples from healthy individuals, isolate lymphocytes and assay at multiple time points using the γ-H2AX assay protocol of the extended RABiT system 2) to demonstrate the practicality and scalability of the extended RABiT system by measuring γ-H2AX yields at multiple time points and generate decay curves and 3) to characterize the effect of demographic characteristics such as age, genotype- ethnicity and race, hormonal influences-gender and environmental factors- alcohol on the baseline endogenous γ-H2AX, radiation induced γ-H2AX response, DNA repair kinetics, repair rate, and residuals. The induction, repair and disappearance of DNA DSB damage was followed microscopically by immunostaining the phosphorylated form of H2AX in unstimulated lymphocytes. Variations in the γ-H2AX decay rate and repair efficiency up to 24 hours post-irradiation are presented.

## Materials and Methods

### Blood collection

All research involving human participants was approved by the Columbia University Medical Center Institutional Review Board (IRB-AAAF3516). Informed written consent, was obtained from the participants. Finger stick blood samples were collected in heparin coated PVC Safe-T-Fill capillaries (RAM Scientific Inc., Yonkers, NY) or MiniCollect 1 cc tube and MiniCollect capillaries (Greiner Bio-one North America Inc, Monroe, NC) from 94 healthy volunteers in the age range 19 to 50 years. Demographic information for gender, age, ethnicity, smoking and alcohol consumption status were recorded for each donor. Alcohol consumption status represents donors that reported consumption of any amount of alcoholic beverages as alcohol consumers and no alcohol consumption as non-alcohol consumers. The females were 63.80% and males 36.17% of the adults recruited (S1 Table). Of the total individuals recruited, majority were White (37.23%), with individuals reported as Other at (24.46%), African American (18.08%) and Asians (13.82%). The non-Hispanic ethnic group was 56% of the total donors. Of the total adults, 62.7% were alcohol consumers of which 12% were reported to be wine drinkers. A majority (95.74%) of the donors were never smokers. Blood samples (30 μl) were pipetted into heparin-coated PVC capillaries (containing 50 μl lymphocyte separation medium (Histopaque-1083; Invitrogen, Eugene, OR) and sealed using Hemato-Seal tube sealing compound (Fisher Scientific, Pittsburgh, PA) as described in [8, 32]. The capillaries were irradiated with γ rays (0, and 4 Gy) using a Gammacell 40 ^137^Cs irradiator (Atomic Energy of Canada, Ltd.). After irradiation, the samples were incubated at 37°C for 30 min to allow for the formation of γ-H2AX foci at the DSB sites. The irradiated blood samples were manually loaded into a custom-designed centrifuge bucket capillary insert.

### Human lymphocyte isolation

To isolate the lymphocytes, the blood samples were spun at 3750 rpm for 5 min to form a distinct lymphocyte band at the interface between the blood plasma and the separation medium. The capillary tubes were cut manually between the lymphocyte band and red blood cell (RBC) pellet as described elsewhere [8, 32]. The isolated lymphocytes and plasma were gently emptied into 96 well tissue culture plate well (Eppendorf, Hauppauge, NY) containing 170 μl RPMI complete medium and placed in the incubator 37°C. At each time point 20 μl aliquot from each respective sample wells were transferred to polycarbonate filter-bottomed multiwell plate (HTS Solubility Filter Plates, Millipore, Billerica, MA) with a pore size of 0.4 μm containing 80 μl phosphate-buffered saline (PBS). To enhance the hydrophilic property of the polycarbonate membranes, the filter plates are pretreated with 25% methanol and washed twice with PBS. The multiwell plate with the isolated lymphocytes was then transferred to the prototype of the extended RABiT system, a robotic plate handling system for automated filtering and liquid handling as described in [33]. As a first step, the positive pressure unit on the gantry system seals the top surface of the plate and applies a positive pressure of 2 PSI for 1.5 s, sufficient to purge the blood plasma and separation medium through the filter plates into the waste system. After the first filtration, the cells are washed by dispensing 100 μl PBS. After the initial wash step, the cells are fixed with 100 μl ice-cold methanol for 10 min. During this time, the filter plate is transferred to a cold surface plate set at −20°C to keep the filter plate cool during the lymphocyte fixation step.

### Immunolabeling γ-H2AX

For the immunodetection of γ-H2AX, the lymphocytes are blocked with 3% bovine serum albumin (BSA; 50 μl) for 30 min followed by exposure to an anti-human γ-H2AX monoclonal antibody (dilution 1:750; ab18311 Abcam Inc., Cambridge, MA) for 1 h at room temperature. Next, the cells are washed five times with 100 μl PBS. To visualize the γ-H2AX foci, the lymphocytes are exposed to a goat anti-mouse Alexa Fluor 555 (AF555) secondary antibody (dilution 1:1000; Invitrogen) for 50 min. After the final wash, the polycarbonate filter bottoms of were detached from the multiwell plate and transferred to an transparent low fluorescence adhesive film (Clear View long lasting packaging tape; Staples, Framingham, MA), sealed with cover slip, mounted on microscopic glass slides using Vectashield DAPI mounting medium (Vector Laboratories, Burlingame, CA). Images for 50–100 cells were captured for each donor at each time point manually (50 ms integration time) with an Olympus epifluorescence microscope (Olympus BX43; Center Valley, PA). Fluorescent images of DAPI-labeled nuclei and AF555-labeled γ-H2AX were captured manually or automated by using NIH Image J Micromanager microscope automation software [34] separately for each dose using a 60 × oil immersion objective (S1 Fig). The images were then analyzed using the RABiT image analysis software [8]. The RABiT image analysis software selects analyzable nucleus based on nuclear conformation and size criteria thereby eliminating abnormal cells for further details on image analysis refer [8].

### Statistical Analysis

Statistical analysis was performed using SigmaPlot 12.0 and Microsoft Excel 2010. The kinetics of γ-H2AX total fluorescence intensity per nucleus was expressed in a non-linear fit function combining linear growth and exponential decay functions. Only samples with complete datasets for all the time points were used for analysis (n = 81). Total γ-H2AX fluorescence at endogenous (0 h), radiation induced (0.5 h) and residual (24 h) levels between each demographic subgroup were analyzed to assess the inter-individual variation in baseline DNA repair levels at 0 h, induction response at 0.5 h and assess the DSB repair efficiency at 24 h (S2 Table). The endogenous γ-H2AX levels (0 h) were subtracted from γ-H2AX levels at 0.5 h from each donor. The resultant means of radiation induced foci (RIY) from each comparison group were statistically evaluated. Similar to the RIF at 0.5 h, the residual γ-H2AX levels were compared between demographic groups. These were calculated by subtracting the baseline γ-H2AX level (0 h) from 24 h γ-H2AX levels for each donor. DNA DSB repair rate (DSBR) for the fast component of repair was calculated for the 2 h period following peak γ-H2AX induction using normalized average pixel value. The resultant means of the data sets at each time points along with standard error of mean (SEM) at 0 h, 0.5 h, 2 h 4 h, 7 h and 24 h are presented in S3 Table. Simple linear regression analysis was applied to investigate relationships between continuous independent variable-age and dependant variable (γ-H2AX levels at each time point or rate of DSB repair). Multiple regression analysis was done to test simultaneously the effect of multiple independent variables (gender, race, ethnicity, alcohol use) on dependant variables (γ-H2AX levels at each time point or rate of DSB repair). Kruskal-Wallis One way analysis of variance on ranks was used to identify variation in yields among the samples classified as per racial group. All P-values were two sided and associations were considered statistically significant at P <0.05. To quantify strength of association between endogenous γ-H2AX level, RIY levels and residual levels, Pearson Product Moment Correlation was used. Correlation coefficients (r) and P <0.05 were used to determine relationship and statistical significance between the pairs.

### Quantitative Modeling

The data on γ-H2AX foci (F) at different time points (T) after irradiation were quantitatively modeled by the following equation, where F_bac_ is the background value prior to irradiation, F_res_ is the residual value remaining at long times (e.g. 24 h) after irradiation, K_prod_ is the constant for induction of foci by radiation, and K_dec_ is the constant for decay of foci after irradiation:

$$F=F_{bac}+F_{res}+K_{prod}\;T_{exp}\;(-K_{dec}\;T)$$ Eq. 1

Various assumptions were applied to the four model parameters (F_bac_, F_res_, K_prod_, and K_dec_). In the most simple and restrictive scenario, each parameter was assumed to have one common value for all analyzed individuals. In contrast, in the least restrictive scenario each parameter was allowed to have a separate value for each individual. In intermediate scenarios, parameter values were assumed to depend on variables such as age, sex, race, alcohol use, or tobacco smoking. The model, using assumptions appropriate for each scenario, was fitted to the data by maximizing the log likelihood using a customized simulated annealing algorithm written in FORTRAN 77.

The relative degree of support provided by the data for each scenario was assessed by the information-theoretic (I-T) approach, using the Akaike information criterion corrected for sample size and parameter number (AICc) [35, 36]. The absolute AICc values contain constants which are specific to the analyzed data. Consequently, inferences are drawn from differences between AICc values produced by different models fitted to the same data. An AICc difference of 6 is generally considered significant because it suggests that support for the tested model is <0.05, compared with support for the best-fitting model among those being compared. In other words, if the AICc for the tested model is 6 or more units greater than the AICc for the best-fitting model, then the support for the tested model is 20 or more fold worse than for the best-fitting model.

## Results

### Variability in the γ-H2AX foci repair kinetics

**Fig 1:**
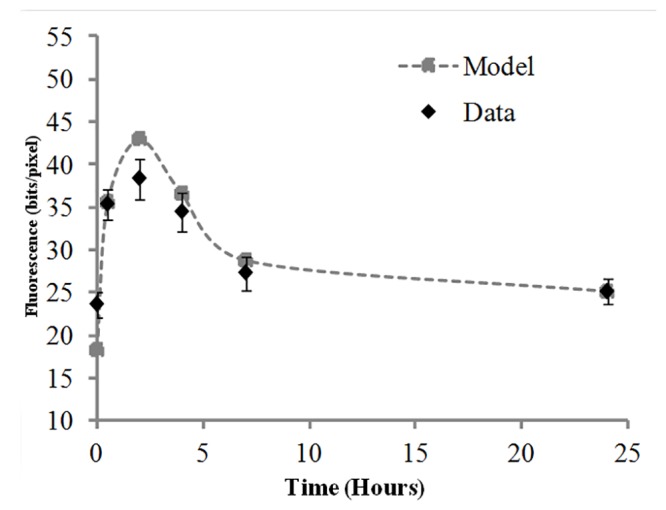
Donor γ-H2AX yields repair kinetics. Experimental data and model fit (from Equation 1) of γ-H2AX yields repair kinetics pooled from 94 donors exposed *ex vivo* to 4 Gy gamma radiation at 0.5 h, 2 h, 4 h, 7 h and 24 h post irradiation. Error bars represent ± SEM.

Initially we characterized the overall γ-H2AX foci induction and decay kinetics in the isolated lymphocytes from the donors following a single 4 Gy gamma dose exposure as shown in Fig. 1. Background corrected average of the total fluorescence obtained from the donors at each time interval of 0 h, 0.5 h, 2 h, 4 h, 7 h, 24 h are plotted with error bars representing standard error of the mean (SEM). The γ-H2AX kinetics followed a biphasic curve, a rapid phase of foci formation followed by an exponential γ-H2AX foci decay phase. Radiation induced γ-H2AX total fluorescent yields (RIY) rapidly increased 0.5 h post radiation and peaked at 2 h with gradual decline over time reaching close to control levels at 24 h post radiation (Fig. 1). Almost 75% of foci disappeared by 7 h (5h post γ-H2AX foci peak formation), while at 24 h post radiation the level of γ-H2AX foci reduced to 10% residual foci. The experimental data were analyzed by a mathematical model (Equation 1 in the Methods section) which allowed the key parameters involved in the foci kinetics to be quantified. The model assumed that a certain background level of foci (F_bac_) was present before irradiation. Due to the radiation effects, additional foci were induced (proportional to the constant K_prod_). These excess foci decayed over time after irradiation (proportional to the constant K_dec_). At long times after irradiation, the foci number asymptotically approached some residual level (F_res_), which could be higher than the pre-irradiation background level. The data indicates inter-individual variability contributing to residuals remaining at 24 h.

**Fig 2:**
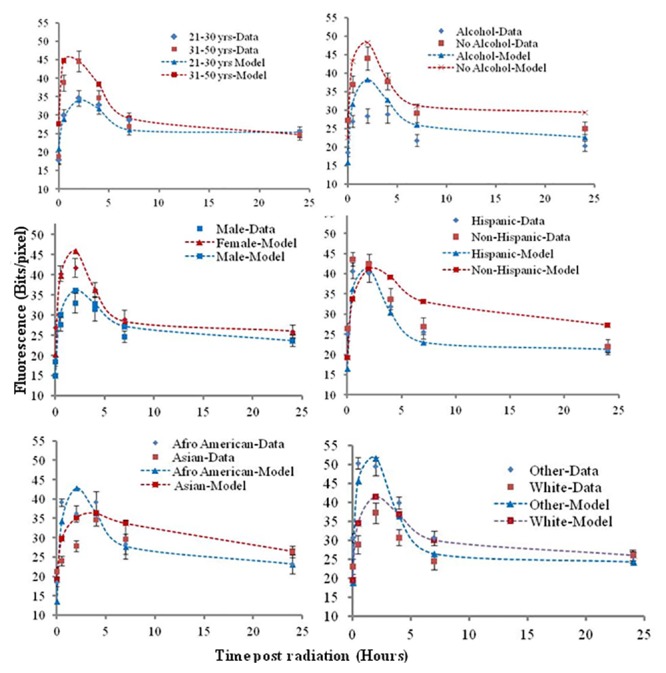
γ-H2AX yields repair kinetics based on demographic groups. (a) Experimental data and model fit (from Equation 1) of γ-H2AX foci repair kinetics at 0.5 h, 2 h, 4 h, 7 h and 24 h post ex vivo gamma irradiation based on (A) age (B) alcohol use (C) gender (D) ethnicity (E) and (F) racial groups. The data obtained is plotted as points with error bars. The predictions from the model are depicted with correspondingly colored dotted lines. Error bars are ±SEM.

We were particularly interested in evaluating the effect of genetic, gender, age and lifestyle factors on the variability in γ-H2AX repair kinetics. For this purpose, the γ-H2AX foci repair kinetics was plotted based on each demographic group as shown in Fig. 2. The donors were sorted based on each group and average of γ-H2AX total fluorescent pixel intensity value from each group at each time point plotted with error bars representing standard error of the mean (SEM). Although the biphasic nature of the γ-H2AX repair kinetics was observed for all the groups, some variability in the endogenous levels, foci induction curve and the exponential decay curve is evident. This variability is described in the sections below.

### Effect of age, race, gender, ethnicity and alcohol use on γ-H2AX endogenous levels

We statistically evaluated the demographic group/s for the effect on variability in endogenous γ-H2AX levels by simple linear and multiple linear regression analysis and mathematical modeling.

**Fig 3:**
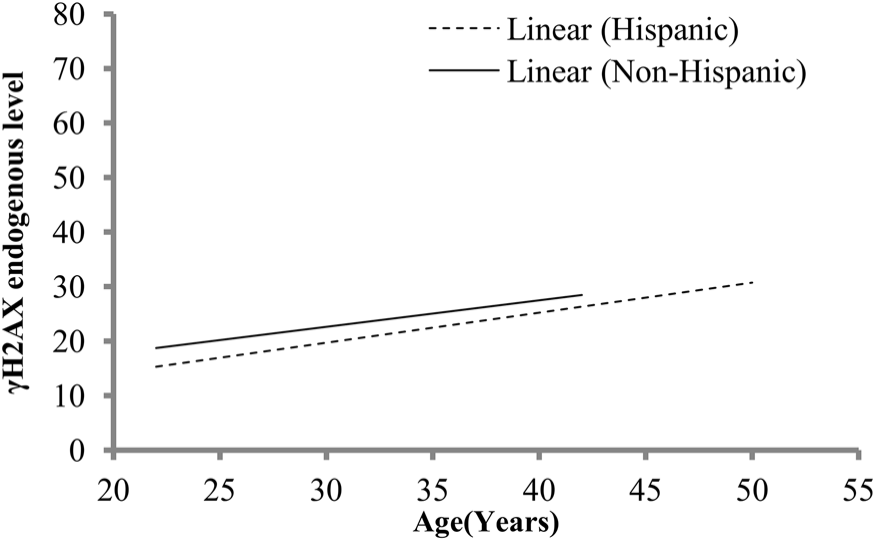
Age, ethnicity and γH2AX endogenous levels. Effect of age on variation in γH2AX endogenous levels on ethnicity is shown with simple linear regression.

Multiple linear regression analysis of the γ-H2AX levels at 0 h of each demographic group (age, gender, race, ethnicity, alcohol use) identified race, ethnicity and alcohol as the influential factors (S4A Table) that were further confirmed by simple linear regression analysis (S4B Table). On identifying race and alcohol group as factors affecting the endogenous γ-H2AX levels, we then further analyzed within race and within alcohol group to determine a specific racial subgroup or if alcohol use was significantly associated with age. Except for weak interaction of age and Whites (P = 0.081), the endogenous levels were independent of a specific racial group and alcohol use. Although total fluorescent γ-H2AX levels in donors was highest for Other race>White>Asian>African American, the variation was not high enough for statistical significance by Kruskal-Wallis One Way Analysis of Variance on Ranks (P = 0.2958). This implies that the inter-individual variability may be contributing to the variation in endogenous levels and/or larger sample size needs to be evaluated for statistical significance. Native and mix race donors were not included in the test since the number of donors for each racial type was less than 5. For both ethnicities, an increase in endogenous levels with age was observed as indicated by inclining regression line (Fig. 3). Consistent with the findings above, a statistically significant interaction between age and alcohol use (P = 0.043), age and race (P = 0.03) was observed but only for Hispanics. A weak interaction of age and gender (P = 0.056) in Hispanics on endogenous levels was also observed. Further, sub-analysis within Hispanics based on race and alcohol use was restricted by the small sample size and hence could not be performed.

The information-theoretic approach applied to the quantitative model described above suggested similar conclusions. Allowing model parameters to vary with age, Hispanic ethnicity, or alcohol use dramatically improved the fit to the data: AICc was reduced by more than 1000 points in each case, compared with the scenario where model parameters were restricted to common values for all individuals. A reduction of AICc of 6 or more units is generally considered to represent a significant improvement in fit, because it suggests that support for the tested model from the data is 20 or more fold better than for the reference model. In this case, AICc reductions were more than 6, suggesting strongly significant effects of age, Hispanic ethnicity, or alcohol use. In contrast, allowing model parameters to vary with gender or race did not improve the fit to the data: AICc was increased by more than 100 points in each case. An increase of AICc of 6 or more units is generally considered to represent a significant decrease in fit quality, because it suggests that support for the tested model from the data is 20 or more fold worse than for the reference model. Consequently, adding extra adjustable parameters to represent potential effects of gender or race made the model fit worse, suggesting that gender or race do not have sufficient effects on this data set to justify excess parameters.

Thus, age was identified as a significant modulator of endogenous γ-H2AX foci variability by both statistical tests and mathematical modeling. Age effects were co-dependent on alcohol use or race. The combination of age/alcohol use and age/race significantly influenced endogenous γ-H2AX foci variability in Hispanics.

### Radiation induced γ-H2AX (RI) response

Following radiation exposure the radiation induced γ-H2AX total fluorescent yields at 0.5 h were computed for each demographic subgroup and compared. The mean γ-H2AX levels obtained at 0.5 h were higher in individuals of 31–50 years (Fig. 2A), donors with no alcohol consumption (Fig. 2B), females (Fig. 2C), Afro-American (Fig. 2E) and Other race (Fig. 2F) but they had no statistical significance by any of the statistical tests. This implies that there was a high amount of variation among individual radiation response within the groups contributing to the visible high γ-H2AX levels in certain groups but lack of statistical significance indicates that overall the radiation induced DNA repair response was similar in the population. Thus the γ-H2AX yields at 0.5 h were independent of race, gender, age, ethnicity with exception of alcohol use. Within the donors that consumed alcohol, further segregation on the type of alcoholic beverage consumed resulted in a group of 12 donors that were wine drinkers alone. To evaluate a trend and relation of the radiation induced γ-H2AX levels at 0.5 h with age, linear regression analysis indicated a trend of linear increase of the γ-H2AX levels with age with no statistical significance (P = 0.09).

In addition to the variability in the γ-H2AX total fluorescent intensity, variability was also observed in the time taken for the foci to reach a maximum level. A rapid increase in foci with a peak at 0.5 h was observed both for Hispanics and Non-Hispanics (Fig. 2D) alone. A later peak at 2 h was noted for donors of all age (Fig. 2A), non alcohol consumers (Fig. 2B), both gender (Fig. 2C), Hispanics (Fig. 2D) and White and donors of Other race (Fig. 2F). In comparison, alcohol consumers (Fig. 2B) and Asians (Fig. 2F) had a relatively slower increase in foci levels that continued through 2 h and peaked at 4 h post radiation. It is interesting to note that the alcohol consumers started with a lower basal γ-H2AX levels than their non-alcohol consuming counterpart.

We performed correlation analysis between endogenous γ-H2AX levels (0 h) and radiation induced γ-H2AX levels (0.5 h) for the entire dataset by Pearson test. A positive and significant correlation with a correlation coefficient of 0.257 and P = 0.020 was obtained. This implied that individuals with higher endogenous levels correlated with higher induction of γ-H2AX post radiation at 0.5 h which indeed was we found for γ-H2AX levels at 0.5 h in females and non-alcohol consumers. Overall, initial radiation induced response observed at 0.5 h was independent of gender, race, ethnicity, age and partly dependant on alcohol use. A variation in the DNA repair induction response was observed with fastest γ-H2AX peak response at 0.5 h when donors were classified based on ethnicity, followed by a peak at 2 h for gender, donors with White and Other race, Hispanics, non alcohol consumers and the donors of all age, and a delayed γ-H2AX peak response for Asians and alcohol consumers with a peak at 4 h. A positive correlation between endogenous and radiation induced γ-H2AX level (0.5 h) was observed for the donors.

### γ-H2AX decay/repair kinetics and effect of gender and ethnicity on residual levels

The γ-H2AX repair kinetics for the healthy donors followed an exponential decay curve (Fig. 1) with the rapid loss of yields from 2 h peak up to 7 h followed by slow, gradual decline of total fluorescent intensity reaching close to baseline levels at 24 h post irradiation. In the decay curves from the demographic groups a similar trend was observed, irrespective of the delayed 4 h γ-H2AX fluorescence peak for alcohol consumers (Fig. 2B) although a steep decrease in fluorescence from 7 h to 24 h was seen for Non-Hispanics (Fig. 2D) and for Asians (Fig. 2E).

**Fig 4:**
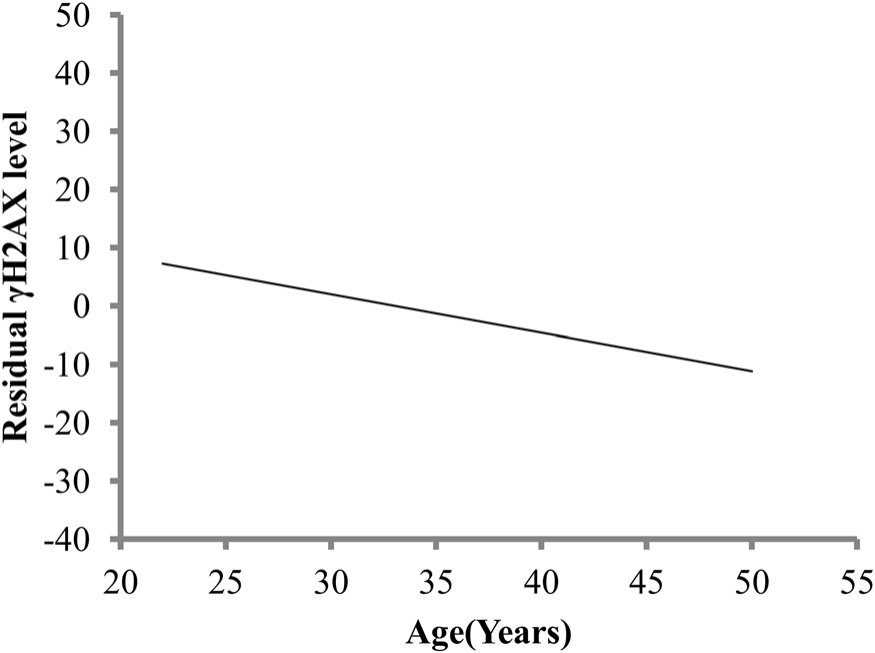
Age and γH2AX foci residual levels. Effect of age on variation in γH2AX foci residual levels at 24 h are shown with simple linear regression.

Recent studies have indicated a possible correlation between radiosensitivity of cells and the residual foci [24, 29, 37]. It is known that radiation induced residual foci may persist even longer than 24 h but not much is known about influence of genotypic, age, gender and lifestyle effects on residual levels. We therefore segregated and analyzed demographic groups in order to identify trends and relations. Association of age with residuals was significant with multiple linear regression analysis (P = 0.046) (S4 Table). In response to age, the residuals levels declined with increasing age as seen by decreasing regression line (Fig. 4). We determined correlation between the two pairs: endogenous levels and residuals, RIY levels and residuals. A negative correlation between γ-H2AX endogenous and residual level with a correlation coefficient of -0.521 and P<0.0001 was observed. Infact, females had higher endogenous γ-H2AX levels and had lower residuals than their male counterparts. No significant correlation of the residuals with RIY levels was observed (P = 0.860).

### Influence of race and tendency of increasing DSB repair (DSBR) rate with age

**Fig 5:**
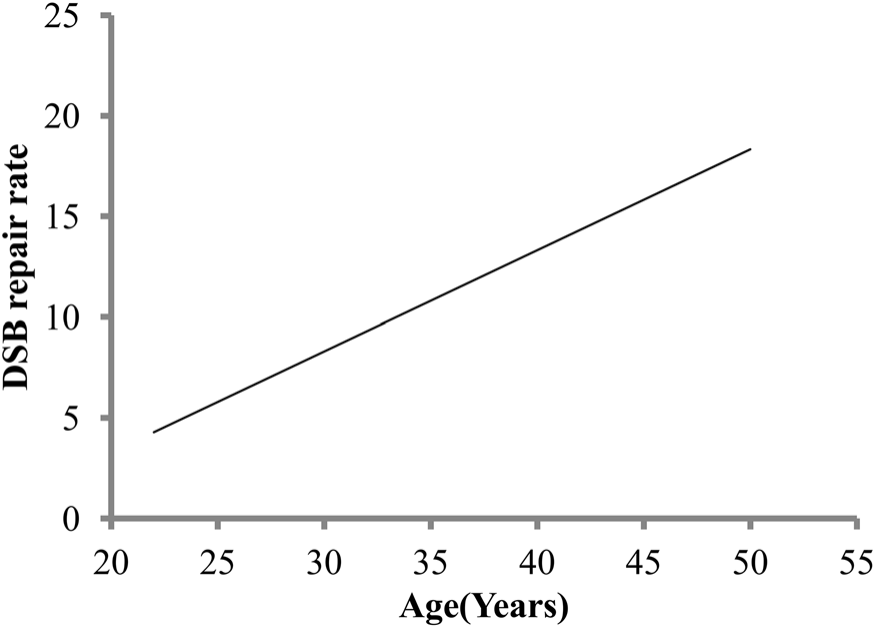
Age and double strand breaks repair (DSBR) rate. Simple linear regression of double strand breaks repair (DSBR) rate shown in response to age (years).

The sensitivity of the microscopic detection of γ-H2AX fluorescence intensity, and the capability to follow the loss of fluorescence by measuring the difference in pixel intensity through the repair kinetics enabled us to calculate rate of DSB repair (DSBR) in our study. DNA DSBR rate for fast component of repair i.e. the first half of the decay curve was determined by the difference at the 2 h period after the γ-H2AX fluorescence levels reached maximum. The obtained DSBR rate was analyzed for age, race, ethnicity, alcohol use and gender. DSBR positively and significantly associated with age as can be seen by inclining regression line (P = 0.025) (Fig. 5). We then analyzed age in combination with other parameter for association with variability in DSBR rate. No significant interactions were found between DSBR rate and ethnicity, alcohol use and gender with age. A statistically significant interaction was found between age and race (P = 0.042). The donors from two major racial groups- Other race and White together with age were then analyzed by multiple regression analysis. We found a statistically significant positive interaction only for White donors and age irrespective of gender (P = 0.007) on DSBR rate. No significant interaction between age and other racial group for DSB rate parameter was detected. To identify potential associations between DSBR rate and induced γ-H2AX response at 0.5 h, we used Pearson correlation and found positive association with a correlation coefficient of 0.634 and P<0.0001.

## Discussion

In this small scale study, we demonstrate scalability and practicality of the extended RABiT system by performing the DNA DSB repair kinetics at multiple time points in unstimulated lymphocytes isolated from normal healthy individuals. Effects of genetic predisposition factors such as race, ethnicity, hormonal influences-gender, behavioral influences such as alcohol use on the known variability of γ-H2AX levels in normal individuals are studied. We identified that the variability in the endogenous γ-H2AX levels is significantly affected by race, ethnicity and alcohol use. A trend of increase in γ-H2AX endogenous levels with age was observed. We show that different donors, independent of age, gender, race, ethnicity or alcohol use respond in a very similar manner to IR exposure. We observed that endogenous levels positively correlated with IR induced γ-H2AX response (0.5 h) and negatively with residual γ-H2AX foci at 24 h. The parameter DSB repair rate was useful measurement for fast component of the DSB repair phase. We show that the rate of fast component of DSB repair is dependent on race and age and independent of gender, ethnicity and alcohol use. A positive correlation between IR induced γ-H2AX response (0.5 h) and DSBR rate was observed. Finally an increase in rate of fast component of DSB repair coincided with lower residuals in older donors.

During many common cellular processes, including DNA replication, cellular senescence and exposure to reactive oxygen species damage to DNA may result in DSB formation and endogenous γ-H2AX foci are formed even in the absence of external DNA damaging agent such as radiation [38]. For biodosimetry purposes in a radiation accident scenario it is evident that the baseline γ-H2AX levels will be unavailable for the exposed individuals. A high variation in base levels between individuals is known [39] and a range of physiological and lifestyle factors is speculated to influence the basal levels. Nevertheless, not much is known about the age, genetic, lifestyle and gender effects on the inter-individual variability in the γ-H2AX endogenous levels. To identify factors affecting this variability we measured the total fluorescent intensity in the unirradiated lymphocytes in the donors recruited from local area using the pre-optimized RABiT γ-H2AX immunofluorescent assay. We identified significant variability in endogenous levels primarily associated with race, ethnicity and alcohol use. A trend of linear increase in endogenous γ-H2AX with age was observed. Our result was in agreement with an ongoing population-based cohort study [19] on Whites in age range (35–83 years) that showed an increase in endogenous γ-H2AX foci linearly with age peaking at ~57 years. A similar observation was reported in a study examining lymphocytes from 21–72 years old [20], incidence of endogenous γ-H2AX foci increased with age until ~50 years old and were considerably higher in age matched Werner syndrome patients. In addition statistical evidence indicated that basal foci variability may arise from the racial differences and influence of lifestyle factors e.g. alcohol such that non-alcohol consumers had high endogenous γ-H2AX levels. While the significance did not hold with interracial analysis due to small sample size (n = 3 to 30), the association highlights the inherent genetic variability in the endogenous γ-H2AX levels. Gender differences in the endogenous levels were also identified with the females having higher endogenous γ-H2AX levels in comparison to males. This result is in contrast to Schurman et.al 2012 [19], they show no significant difference in γ-H2AX endogenous foci levels in the peripheral blood mononuclear cells (PBMCs) derived from leukapheresis in donors (35–83 years). Only less than 10 donors in their study were in the age group of below 50 years for reasonable comparison with results in our study. Moreover, variation in the methods of blood collection and treatment of samples are factors that may contribute to variability in between laboratories. Endogenous levels were also independent of age/gender and this observation was further confirmed from the sub-analysis in females where the higher endogenous γ-H2AX levels were found to be age independent. Our results was in concurrence with previous report [39] where no effect of these factors on the basal levels of γ-H2AX was identified. Furthermore we also found that age and race/alcohol use was significantly associated with endogenous levels only in Hispanics. This indicates that ethnicity may be a confounding factor influencing γ-H2AX levels with age and race/alcohol use. Further studies are needed on a larger ethnic group to confirm this observation. In addition, diet as a significant contributor in modulation of DNA repair functions has been reported [16, 17] and could be a major contributor.

Despite the variability in endogenous levels of γ-H2AX, the donor response to radiation at 0.5 h was independent of age, gender, race, ethnicity and alcohol use. Similar observations were reported in studies where donor radiation response was found to be independent of age and gender [4, 20, 40]. Previous report by Sedelnikova et. al [20] suggest an age independent response of the number of IR induced γ-H2AX foci at 0.5 h and decrease in their numbers 8 h post radiation, consistent with this observation we show age independence of IR induced γ-H2AX foci 0.5 h post IR and also at 7 h post radiation. In agreement with a study that showed correlation between higher γ-H2AX endogenous levels and increased initial and/or residual DNA damage level after irradiation *in vitro* [28], we observed an increase in the initial damage response in donors with higher endogenous levels. Our data indicated a negative correlation of the endogenous levels with the residual foci.

We observed biphasic kinetics of DSB repair in this study and the biphasic model fit closely with our data. These patterns were incorporated into our quantitative formalism (Equation 1 in the Methods section), which modeled induction of foci by radiation, decay of these radiation-induced foci over time, and persistence of some residual foci even at long times (e.g. 24 h) after exposure. Consistent with these assumptions and previous experimental studies, we observed a rapid increase in foci at 0.5 h post radiation. This rise was independent of age, gender, ethnicity, race and alcohol use. This increase in foci continued with formation of a peak 2 h post radiation for majority of demographic groups. In agreement with this study, previous studies conducted using human lymphocytes found maximum γ-H2AX foci induction at 1–2 h post exposure [12, 41]. Delayed foci induction response was observed for Asians and donors with alcohol use with peak formed at 4 h. A similar delayed DNA damage response was reported in a rat alcohol genotoxicity study, where the data on late RAD51, 53BP1 and γ-H2AX foci formation indicated that the triggered DNA damage response to DSB lesions was not immediate and was around 3–4 h post alcohol derived acetylaldehyde byproduct treatment [42]. It should be noted that in addition to the alcohol use, an added genotoxicity agent such as radiation resulted in a similar delay in the DNA repair response in our study as for rats exposed to alcohol alone in the alcohol genotoxicity study. It is therefore possible that behavioral factors such as alcohol use may have an underlying effect on the DNA repair response and radiation effect may necessarily not be additive; albeit these factors are important to explain the variability arising in the repair kinetics. Besides the behavioral factors, genetic defects in the DNA damage sensory protein pathway are known to slow DNA repair response seen early on after IR [43, 44].

In addition to the variation in the IR induced foci response, variability in the decay kinetics was also observed. The presence of two phases in the decay kinetics of DNA DSB repair is widely established, a rapid phase lasting few hours followed by a slower phase that last several hours to days [45, 46]. Approximately 80% of DNA breaks are repaired during the fast component of repair process and the remaining ~20% repair more slowly [45, 46]. These remaining breaks depending on the complexity of damage repair over a varying period of time [47]. We found about 25% of γ-H2AX residual foci remaining at 7 hours post IR consistent with findings that showed slow γ-H2AX repair kinetics in lymphocytes from healthy donors [4]. We show that the rate of the fast component of the decay kinetics is significantly higher for older individuals that coincide with lower residuals. A study on recruitment of repair proteins in human lymphocytes and fibroblasts from normal and Werner syndrome donors reported a decrease in speed of repair protein recruitment relative to aging and that a slower DSB repair resulted in accumulation of persistent DNA lesions [20]. In contrast, donors in our study irrespective of age, showed a similar response for induction of foci and for maximum recruitment of γ-H2AX peaking at 2 h post radiation. Moreover, the rate of fast component of DSB repair significantly increased with aging with a statistically significant corresponding decrease in residual levels for older donors.

Estimation of DSB repair rate from decay kinetics of γ-H2AX using sensitive immunofluorescent technique as in this study can be a useful parameter to evaluate foci kinetics and cellular radiosensitivity. A significant positive correlation between DSB repair rate and radiation induced γ-H2AX response (P<0.0001) was observed. Both DSBR rate and RIY increased with increasing age. This was seen in the older donors (31–50 yrs) who although started off with higher γ-H2AX foci peak at 2 h than the younger donor group (21–30 yrs), a rapid repair rate for the fast component of DNA decay curve resulted in comparable γ-H2AX yields at 7 h and 24 h for both groups. These results are in contrast with those of Garm et. al. (2012) [40] who reported a statistically non-significant decrease in γ-H2AX response and DSB repair with increasing age in human PBMCs. The reason for discrepancy may reside in the characteristics of their study population which consisted of population-based sample of twins in age group of 40–77 years with mean age of 55.6 years, in comparison individuals in our study were a sample from population of different genetic background and much younger with a mean age of 31 years. In another small study on DSB repair in unstimulated lymphocytes from 20 healthy donors aged (23–78 years) irradiated with 30 Gy X-rays, gender specific pattern in DSB repair suggested less efficient repair in women aged <65 years [21]. In contrast, no significant gender based differences was observed in DSB repair rate in our study for individuals between 21–50 years of age. Moreover, the type of radiation, dose received, irradiation conditions and assay type and protocol used to detect DSB repair could also have an effect on the response.

Evidence from several studies suggest existence of defects in genetic repair mechanisms, complexity [48], or conformational inaccessibility of foci due to location within highly condensed heterochromatin [44] resulting in less accessibility for repair proteins to repair and thus retention of unrepaired foci. In addition, to the nature of the lesions, genetic defect or absence of activities of ATM and Artemis endonuclease activity required for the slow component repair may affect the repair rate as shown by studies with cell lines lacking ATM, Artemis and ATM-dependent signaling proteins (e.g., 53BP1). Given the heterogenity in the DSB repair response, chromosome and radiotherapeutic data suggest that only ~5% of the healthy population has deficient DSB repair capability [49]. Studies indicate that the rate of foci loss and presence of residual foci has been correlated with cellular radiosensitivity [24, 29, 50, 51], thus detection of a small number of persisting DSBs is important [46]. The residual levels for the donors in our study were largely influenced by age. Although the residual levels were higher for males, combined analysis of age and gender was significantly associated with residual levels only for females (P = 0.013). Analysis of IR induced foci across different aged mice for both males and females demonstrated that tissues of younger animals were more suspectible to IR induced damage and had higher induction and persistence of γ-H2AX foci [52]. This observation was partially correlated with high rate of cellular proliferation and expression of DNA repair proteins and authors suggested age related predisposition to radiation effects. Although donors in our study had a similar IR response irrespective of age, donors in age group 21–30 years and non-Hispanics had significantly higher residuals.

We found age as a modulator of DSB γ-H2AX repair kinetics. An increase in endogenous foci levels, a trend of increase in RIF γ-H2AX levels and a significant decrease in the residual foci levels with age was observed. Interestingly, DSBR rate for the fast component of repair increased with age. Studies investigating dependence of aging and γ-H2AX formation remain inconclusive. Evidence in the literature from studies on aging and senescing cell lines of epithelial and fibroblastic origin and mice, show an increase in γ-H2AX foci with age [20, 53–55]. Increasing γ-H2AX levels in healthy subjects of different age groups lead [20, 56] these investigators to conclude that aging may cause more DSB and/or alter the functional capacity of human cells to repair DSB resulting in age related decline in DSB repair efficiency and fidelity. In contrast, Firsanov et.al [57], conclude that the dynamics of γ-H2AX formation and kinetics is independent of age. A possible explanation for the discrepancy can be attributed to the cell type, inter-individual variation, younger group (21–50 years with mean age of 31 years) analyzed in our study compared to studies with donors in the age group up to 83 years.

## Summary

Analysis of variability in DNA repair kinetics in a normal mixed population has the potential to extend our knowledge on individual radiosensitivity and variation in inter-individual endogenous γ-H2AX level, information unavailable yet useful for triage decision at the site of nuclear incidents. By closely evaluating interactions of genetic, lifestyle, aging and other factors with DNA DSB repair in donor study, understanding of molecular processes of diseases associated with DNA repair is expanded. We identified significant associations of age, ethnicity, race, gender, and alcohol use with variation in γ-H2AX total fluorescent intensity endogenous and residual levels. Variation in endogenous levels was dependant on alcohol use, ethnicity and age. An increase in the endogenous levels was positively correlated with age. The IR response at 0.5 h was independent of factors studied. Understanding the response to IR by individuals is important for minimizing side effects during radiotherapy. An increase in DSB repair rate of the fast component in individuals of age group 31–50 years coincided with reduced residuals at 24 h. There is now a great deal of interest in mechanistic biomarkers that could ultimately lead to predictive or preventive strategies and also in the context of potential individualized biomarkers for cancer therapy. Until now, there has been no corresponding high-throughput technology for assaying global DSB repair kinetics as developed at the Center for High-Throughput Minimally Invasice Radiation Biodosimetry [33] with demonstration of its practicality and scalability as in this study. Having the technology for a high throughput, inexpensive assay for global DSB repair would allow a new approach—DSB-repair association studies using fingerstick volumes of blood. Assessing the kinetics of a repair protein as in the present small scale study provides a practical, rapid, high-throughput, and inexpensive tool for assessing global DSB repair capacity on an individual-by-individual basis. Potential applications are for epidemiological studies relating to cancer therapy strategies, and also to facilitate development of preventive strategies for a variety of diseases—either in standalone epidemiological studies, or to complement molecularly-based association studies.

## Supporting Information

S1 Fig γ-H2AX expression in human lymphocyte. Representative γ-H2AX staining in isolated lymphocytes irradiated with 4 Gy visualized with Alexa Fluor 555 and for cells fixed at time points 0 h, 0.5 h, 2 h, 4 h, 7 h, 24 h post irradiation. A potential confounder for RABiT imaging analyses is rejection of abnormal cells, an effect of radiation-induced apoptosis that may result in possible bias of γ-H2AX measurements in aging cells. Although presence of abnormal cells was not largely different among the cells captured in two age group cells upto 24 h post irradiation it is recognized as a valid concern for later time points.
(TIF)

S1 Table Demographic details of the recruited donors for the study. (PDF)

S2 Table Raw data for the γ-H2AX kinetics demographic study of the recruited donors for the time points 0 h, 0.5 h, 2 h, 4 h, 7 h and 24 h. (PDF)

S3 Table γH2AX data values are presented as Mean ± SEM at time points of 0 h, 0.5 h and 24 h for each of the demographic groups. (PDF)

S4 Table The effect of age, ethnicity, race and alcohol use on variation in DSB γH2AX repair kinetics at 0 h, 0.5 h and 24 h by A. **Multiple linear regression analysis B. Simple linear regression** analysis for parameters that were significant with multiple regression analysis is presented. Significant P< 0.05.
(PDF)
