# Correction: High Throughput Measurement of γH2AX DSB Repair Kinetics in a Healthy Human Population

**DOI:** 10.1371/journal.pone.0131620

**Published:** 2015-06-22

**Authors:** Preety M. Sharma, Brian Ponnaiya, Maria Taveras, Igor Shuryak, Helen Turner, David J. Brenner

In the Quantitative Modeling section of the Materials and Methods, there is an error in the first equation. Please view the complete, correct equation here:

$$\text{F}=F_{bac}+F_{res}+K_{prod}\;T\;exp\left(-K_{dec}\;T\right)$$
