# Supplementary figures and images for: High Throughput Measurement of γH2AX DSB Repair Kinetics in a Healthy Human Population
Source: PLoS One. 2015 Mar 20;10(3):e0121083. doi: 10.1371/journal.pone.0121083 (PMC4368624; doi:10.1371/journal.pone.0121083)

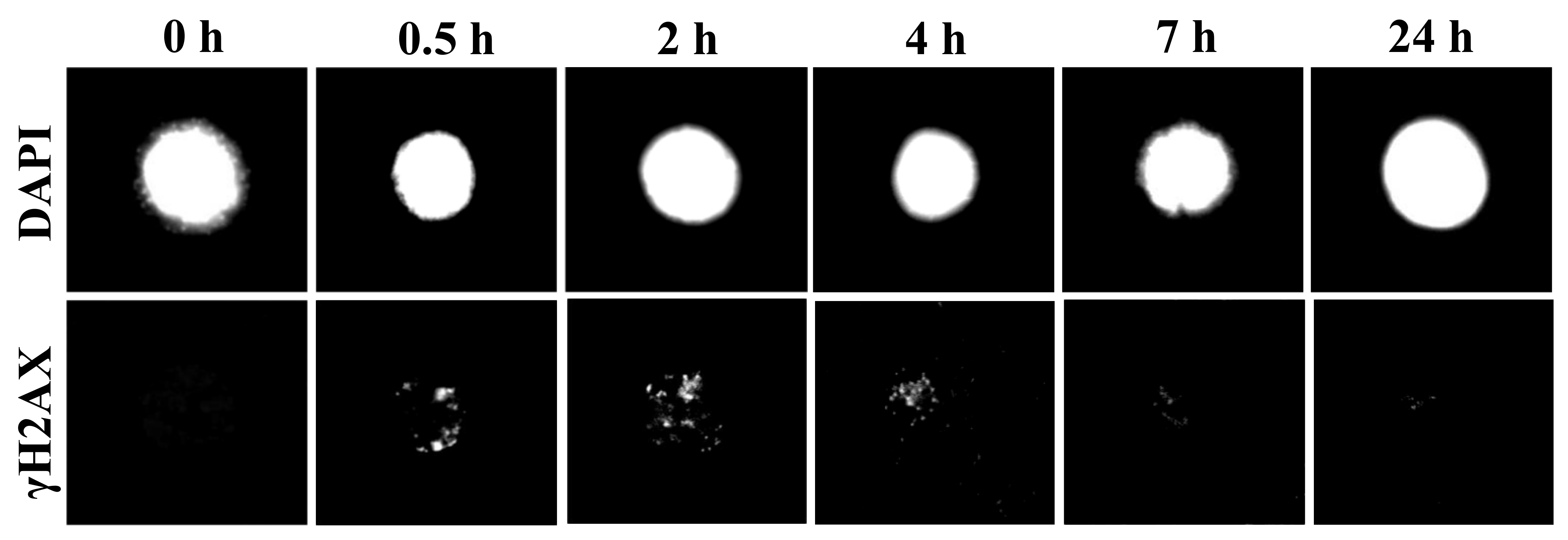

Supplement: S1 Fig — Representative γ-H2AX staining in isolated lymphocytes irradiated with 4 Gy visualized with Alexa Fluor 555 and for cells fixed at time points 0 h, 0.5 h, 2 h, 4 h, 7 h, 24 h post irradiation. A potential confounder for RABiT imaging analyses is rejection of abnormal cells, an effect of radiation-induced apoptosis that may result in possible bias of γ-H2AX measurements in aging cells. Although presence of abnormal cells was not largely different among the cells captured in two age group cells upto 24 h post irradiation it is recognized as a valid concern for later time points. (TIF) [file pone.0121083.s001.tif]
