# Supplementary material for: High Throughput Measurement of γH2AX DSB Repair Kinetics in a Healthy Human Population
Source: PLoS One. 2015 Mar 20;10(3):e0121083. doi: 10.1371/journal.pone.0121083 (PMC4368624; doi:10.1371/journal.pone.0121083)
Supplement: S1 Table — (PDF) [file pone.0121083.s002.pdf]

S1_Table: Demographic details of the recruited donors for the study.

Variables	Number	%
**Total population**	94	
**Age (years)**		
21-30	50	53.19
31-50	44	46.8
**Sex**		
Male	34	36.17
Female	60	63.82
**Ethnicity**		
Hispanic	38	40.42
Non-Hispanic	56	59.57
**Race**		
African American	17	18.08
White	35	37.23
Asian	13	13.82
Other	23	24.46
Native	3	3.19
Mix	3	3.19
**Alcohol consumption**		
Yes	59	62.766
Wine alone	12	12.76
Wine and others	47	50
No	35	37.23
