# Supplementary material for: High Throughput Measurement of γH2AX DSB Repair Kinetics in a Healthy Human Population
Source: PLoS One. 2015 Mar 20;10(3):e0121083. doi: 10.1371/journal.pone.0121083 (PMC4368624; doi:10.1371/journal.pone.0121083)
Supplement: S2 Table — (PDF) [file pone.0121083.s003.pdf]

S2_Table: Raw data for the γ-H2AX kinetics demographic study of the recruited donors for the time points 0 h, 0.5 h, 2 h, 4 h, 7 h and 24 h.

Age	Gender	Race	Ethnicity	Alcohol consumption	0 h	0.5 h	2 h	4 h	7 h	24 h
26	Female	White	Non-Hispanic	Yes	23.06	26.28	24.23	35.46	26.43	27.48
32	Female	Others	Hispanic	Yes	31.43	40.92	14.13	28.45	40.80	29.96
31	Male	Others	Hispanic	Yes	12.99	33.89	38.09	52.98	28.30	44.22
25	Male	Afro-American	Non-Hispanic	Yes	3.41	29.70	40.56	48.63	19.96	26.08
29	Female	Afro-American	Non-Hispanic	Yes	40.38	37.91	35.16	52.71	31.38	36.11
33	Female	Asian	Non-Hispanic	No	25.47	32.83	38.46	56.32	40.17	41.10
26	Male	White	Non-Hispanic	Yes	14.34	32.56	40.64	47.46	25.60	25.08
31	Female	White	Non-Hispanic	Yes	28.10	33.22	30.18	41.73	34.72	32.52
23	Female	White	Non-Hispanic	Yes	15.15	38.56	34.87	39.30	37.12	34.24
26	Male	White	Non-Hispanic	No	11.55	26.32	26.30	67.55	46.15	39.49
30	Male	Asian	Non-Hispanic	Yes	11.60	15.21	39.15	61.73	37.09	26.94
25	Male	White	Non-Hispanic	Yes	15.55	30.03	40.30	36.68	25.43	21.44
45	Female	Others	Hispanic	No	25.49	24.71	22.56	32.26	36.26	21.40
28	Male	Afro-American	Non-Hispanic	Yes	23.02	16.79	39.50	34.59	42.04	21.52
23	Female	Asian	Non-Hispanic	Yes	19.68	34.58	41.28	41.73	31.79	29.66
26	Male	Asian	Non-Hispanic	Yes	19.62	18.47	41.68	43.64	44.07	37.03
26	Female	Asian	Non-Hispanic	Yes	25.12	31.70	14.62	26.62	13.16	18.46
30	Male	White	Non-Hispanic	No	30.97	43.94	42.78	15.44	7.46	13.78
48	Female	Others	Hispanic	No	75.49	108.91	135.99	45.16	28.93	24.72
47	Female	Others	Hispanic	No	26.48	29.38	55.41	37.98	39.45	39.02
30	Male	White	Non-Hispanic	Yes	9.07	18.84	64.18	22.35	35.45	48.04
25	Female	Others	Hispanic	No	24.52	46.26	89.79	57.32	60.03	50.58
34	Female	Others	Hispanic	No	67.79	59.53	35.14	54.49	47.38	45.57
28	Female	White	Non-Hispanic	Yes	35.75	38.85	124.46	52.76	53.80	44.44
22	Female	Asian	Non-Hispanic	No	35.00	38.08	55.92	55.05	50.75	45.73
23	Female	White	Non-Hispanic	Yes	42.63	46.80	57.55	52.52	33.38	65.33
35	Female	Others	Hispanic	Yes	40.01	62.75	101.85	51.07	47.73	45.13
29	Female	White	Non-Hispanic	Yes	67.84	61.96	52.24	58.79	46.22	50.39
28	Male	Afro-American	Non-Hispanic	Yes	52.35	97.16	104.64	69.38	34.50	40.05
36	Female	White	Non-Hispanic	No	37.24	51.76	109.96	30.41	39.15	55.94
38	Female	White	Non-Hispanic	Yes	33.18	61.11	56.97	57.54	27.41	34.07
27	Male	White	Non-Hispanic	No	53.75	74.28	90.59	82.73	83.81	54.35
22	Female	Others	Hispanic	No	21.08	86.93	53.57	42.17	36.11	42.05
31	Female	Others	Hispanic	No	57.64	98.68	101.24	86.72	68.78	57.99
42	Female	Afro-American	Non-Hispanic	No	46.89	86.40	67.71	61.64	47.71	20.59
41	Female	Others	Hispanic	Yes	45.21	97.40	87.29	80.32	33.16	16.91
50	Female	Others	Hispanic	No	65.61	144.22	120.33	102.77	46.32	26.02

43	Female	Others	Hispanic	Yes	3.33	28.78	2.37	3.45	4.25	3.65
25	Female	Afro-American	Non-Hispanic	Yes	3.47	16.73	1.79	2.31	14.64	1.10
30	Female	Others	Hispanic	Yes	4.36	17.93	2.57	4.46	3.44	4.07
44	Female	Native	Hispanic	Yes	3.35	13.85	3.65	3.69	32.07	20.08
40	Female	Afro-American	Non-Hispanic	No	3.09	30.32	46.59	51.45	32.32	27.30
33	Female	Afro-American	Non-Hispanic	Yes	3.48	46.08	30.13	51.70	28.19	25.61
26	Male	Afro-American	Non-Hispanic	Yes	3.94	52.41	27.76	61.48	43.38	35.42
31	Female	Others	Hispanic	No	26.50	30.19	16.70	20.22	7.20	4.65
30	Male	White	Non-Hispanic	Yes	33.29	32.54	29.24	19.13	9.08	8.28
34	Female	Afro-American	Hispanic	Yes	31.33	25.36	21.26	9.80		7.82
31	Male	White	Hispanic	No	18.27	19.43	31.47	10.38	7.94	8.83
46	Male	Native	Hispanic	No	9.12	8.91	35.11	10.98	19.14	14.90
50	Female	Others	Hispanic	No	20.93	16.54	28.86	19.77	13.01	14.35
26	Male	White	Hispanic	Yes	11.69	24.03	33.28	17.21	10.38	23.83
41	Female	Others	Hispanic	No	6.19	10.13	11.85	6.16	5.94	0.99
31	Female	White	Hispanic	Yes	10.30	13.63	9.25	14.19	6.64	1.76
28	Female	Afro-American	Hispanic	Yes	10.83	12.35	9.81	13.80	10.28	0.75
29	Female	Others	Hispanic	No	14.10	1.14	12.47	13.93	21.06	9.62
46	Male	Afro-American	Hispanic	Yes	4.31	1.60	11.86	13.89	16.68	14.76
32	Female	Others	Hispanic	No	8.97	9.62	9.79	8.34	19.08	14.36
37	Female	Others	Hispanic	Yes	9.10	15.95	9.82	13.24	17.22	7.25
23	Male	White	Non-Hispanic	Yes	23.35	26.19	33.13	39.22	17.52	19.94
24	Female	White	Non-Hispanic	No	12.72	18.21	18.45	42.85	9.01	17.02
26	Female	White	Non-Hispanic	Yes	14.18	7.36	8.83	7.70	7.08	10.47
26	Male	White	Non-Hispanic	Yes	16.27	15.03	14.84	23.86	5.67	16.43
26	Female	Afro-American	Hispanic	No	14.03	21.54	22.37	22.97	12.81	10.97
24	Male	Mix	Non-Hispanic	Yes	7.64	25.82	23.67	25.96	8.44	9.90
27	Female	White	Non-Hispanic	Yes	9.20	17.22	23.26	23.23	7.77	10.23
30	Male	Asian	Non-Hispanic	No	21.99	22.54	21.11	16.32	17.14	22.68
27	Male	White	Non-Hispanic	No	20.83	14.39	31.22	19.43	17.27	23.56
36	Male	Asian	Non-Hispanic	No	22.96	30.72	19.70	42.69	21.94	27.36
23	Male	Afro-American	Non-Hispanic	Yes	20.20	30.41	21.31	13.84	24.17	35.63
41	Male	Others	Hispanic	Yes	13.13	17.67	8.75	16.61	12.53	4.49
26	Male	White	Non-Hispanic	Yes	10.95	16.43	15.33	16.93	5.94	7.50
31	Female	Mix	Non-Hispanic	Yes	10.38	9.32	10.49	7.95	12.98	5.58
24	Female	White	Non-Hispanic	Yes	13.35	10.70	9.66	5.91	13.35	8.64
24	Female	White	Non-Hispanic	Yes	9.75	16.18	7.56	5.43	6.41	4.80
25	Female	White	Non-Hispanic	Yes	23.81	6.81	6.86	8.55	15.34	20.52
24	Male	White	Non-Hispanic	Yes	12.81	4.36	3.29	3.74	12.96	14.61
23	Male	Asian	Non-Hispanic	Yes	16.04	9.39	4.85	3.87	14.80	8.02
23	Female	White	Non-Hispanic	Yes	12.32	13.40	8.57	9.26	21.97	12.19

42	Male	Asian	Non-Hispanic	Yes	13.48	17.54	15.14	5.72	26.57	9.76
23	Male	Mix	Hispanic	Yes	7.99	7.85	12.53	5.28	6.73	5.31
32	Male	Afro-American	Hispanic	Yes	9.40	12.98	9.74	9.56	11.79	7.90
