# Supplementary material for: High Throughput Measurement of γH2AX DSB Repair Kinetics in a Healthy Human Population
Source: PLoS One. 2015 Mar 20;10(3):e0121083. doi: 10.1371/journal.pone.0121083 (PMC4368624; doi:10.1371/journal.pone.0121083)
Supplement: S3 Table — (PDF) [file pone.0121083.s004.pdf]

S3_Table: γH2AX data values are presented as Mean ± SEM at time points of 0 h, 0.5 h and 24 h for each of the demographic groups.

Variables	Groups	Baseline (0 h) γH2AX	Radiation induced (0.5 h) γH2AX	Residual (24 h) γH2AX
**Age (Years)**	20-30	20.09±2.01	28.34±2.95	24.25±2.32
	31-50	24.9±3.41	38.95±5.73	22.25±2.72
**Gender**	Female	25.21±2.67	37.32±4.32	24.06±2.48
	Male	17.37±2.07	25.856±3.42	22.41±2.38
**Race**	Others	28.58±4.80	46.73±8.53	24.14±3.96
	White	22.37±2.59	28.01±3.22	25.17±3.13
	Afro American	18±4.42	34.51±6.92	20.77±3.37
	Asian	21.09±2.13	25.1±3.06	26.6±3.95
**Ethnicity**	Hispanic	22.84±3.52	35.255±6.23	19.497±2.91
	NonHispanic	21.636±2.05	30.886±2.849	25.968±2.15
**Alcohol status**	Alcohol	18.61±1.915	27.74±2.77	21.15±2.08
	No-alcohol	28.73±3.69	42.35±6.59	27.67±3.13
