# Supplementary material for: High Throughput Measurement of γH2AX DSB Repair Kinetics in a Healthy Human Population
Source: PLoS One. 2015 Mar 20;10(3):e0121083. doi: 10.1371/journal.pone.0121083 (PMC4368624; doi:10.1371/journal.pone.0121083)
Supplement: S4 Table — Multiple linear regression analysis B. Simple linear regression analysis for parameters that were significant with multiple regression analysis is presented. Significant P< 0.05. (PDF) [file pone.0121083.s005.pdf]

S4_Table: The effect of age, ethnicity, race and alcohol use on variation in DSB γH2AX repair kinetics at 0 h, 0.5 h and 24 h by A. Multiple linear regression analysis B. Simple linear regression analysis for parameters that were significant with multiple regression analysis is presented. Significant P< 0.05.

A. Multiple linear regression analysis

**Time point**	**R**	**Rsqr**	**Adj Rsqr**	**Variables with significant P value**		
**0 h**	0.444	0.197	0.144	Race (P=0.027)	Ethnicity (*P=0.063*)	Alcohol (P=0.047)
**0.5 h**	0.250	0.0625	0.00005	None		
**24 h**	0.362	0.131	0.0732	Age (P=0.046)		

B. Simple linear regression analysis

**Time point**	**R**	**Rsqr**	**Adj Rsqr**	**Variables with significant P value**
**0 h**	0.281	0.0790	0.0674	Race (P=0.011)
	0.290	0.0842	0.0726	Alcohol (P=0.009)
**0.5 h**	None			
**24 h**	0.314	0.0989	0.0875	Age (P=0.004)
